# Main factors associated with foot-and-mouth disease virus infection during the 2001 FMD epidemic in Uruguay

**DOI:** 10.3389/fvets.2023.1070188

**Published:** 2023-02-02

**Authors:** María V. Iriarte, José L. Gonzáles, Eduardo de Freitas Costa, Andrés D. Gil, Mart C. M. de Jong

**Affiliations:** ^1^Quantitative Veterinary Epidemiology, Wageningen University, Wageningen, Netherlands; ^2^Department of Epidemiology, Official Veterinary Services, Ministry of Livestock, Agriculture and Fisheries of Uruguay, Montevideo, Uruguay; ^3^Department of Epidemiology, Bioinformatics and Animal Models, Wageningen Bioveterinary, Lelystad, Netherlands; ^4^Facultad de Veterinaria, Universidad de la República del Uruguay, Montevideo, Uruguay

**Keywords:** foot-and-mouth disease (FMD), risk factors, epidemic, high risk period, FMDV infection

## Abstract

Large epidemics provide the opportunity to understand the epidemiology of diseases under the specific conditions of the affected population. Whilst foot-and-mouth disease (FMD) epidemics have been extensively studied in developed countries, epidemics in developing countries have been sparsely studied. Here we address this limitation by systematically studying the 2001 epidemic in Uruguay where a total of 2,057 farms were affected. The objective of this study was to identify the risk factors (RF) associated with infection and spread of the virus within the country. The epidemic was divided into four periods: (1) the high-risk period (HRP) which was the period between the FMD virus introduction and detection of the index case; (2) the local control measures period (LCM) which encompassed the first control measures implemented before mass vaccination was adopted; (3) the first mass vaccination, and (4) the second mass vaccination round. A stochastic model was developed to estimate the time of initial infection for each of the affected farms. Our analyses indicated that during the HRP around 242 farms were probably already infected. In this period, a higher probability of infection was associated with: (1) animal movements [OR: 1.57 (95% CI: 1.19–2.06)]; (2) farms that combined livestock with crop production [OR: 1.93 (95% CI: 1.43–2.60)]; (3) large and medium farms compared to small farms (this difference was dependent on regional herd density); (4) the geographical location. Keeping cattle only (vs farms that kept also sheep) was a significant RF during the subsequent epidemic period (LCM), and remained as RF, together with large farms, for the entire epidemic. We further explored the RF associated with FMDV infection in farms that raised cattle by fitting another model to a data subset. We found that dairy farms had a higher probability of FMDV infection than beef farms during the HRP [OR: 1.81 (95% CI: 1.12–2.83)], and remained as RF until the end of the first round of vaccination. The delay in the detection of the index case associated with unrestricted animal movements during the HRP may have contributed to this large epidemic. This study contributes to the knowledge of FMD epidemiology in extensive production systems.

## 1. Introduction

Foot-and-mouth disease (FMD) is a transmissible viral disease that globally affects cloven-hoofed animals, including domesticated and wildlife species (1, 2). FMD continues to be one of the most important diseases of livestock worldwide as it limits international trade in animals and animal products, causes productivity losses and is costly to control (3). In short, a foot-and-mouth disease virus (FMDV) outbreak in a FMDV free country that produces livestock for export has enormous economic consequences for that country. This is certainly true in the case of Uruguay, which is recognized as a FMD free country with vaccination by the World Organization for Animal Health (WOAH, formally known as OIE), and for which the exports of livestock products represent around the 30% of the country's total value exports (4).

During the first decade of the 21st century, FMDV has caused concern worldwide as it has appeared in previously FMDV-free territories. This occurred in the United Kingdom (UK) in 2001, which led to introductions in other countries of the Europe Union (EU), such as France, Ireland and The Netherlands (5, 6). Other large epidemics also occurred in free countries in South America, one of those countries being Uruguay, which was considered a FMDV-free country without vaccination since 1996 by the WOAH. However, in October 2000 Uruguay experienced a minor and localized outbreak of FMDV type O, which was controlled by stamping out, movement restrictions and disinfection (7). The country achieved eradication and regained the FMDV-free status without vaccination by WOAH in January 2001. In April 2001 a new epidemic of FMDV type A started in Uruguay. This epidemic followed an ongoing FMDV type A epidemic in Argentina, which started some time before January 2001 (8). During this epidemic, 2,057 farms were affected in Uruguay.

Large epidemics provide the opportunity to understand the epidemiology of diseases under the specific conditions of the affected population. The identification of disease risk factors from data generated during epidemics can be useful to generate recommendations for future outbreaks and to guide effective surveillance systems as well.

Several researchers have reported risk factors associated with FMDV infection. Cattle herds and large farms were more strongly associated with outbreaks during the 2001 epidemic in UK (9). The structure and density of the production sector in a country are also likely to be risk factors for infection. For example, most infected herds in the 2001 epidemic in the Netherlands were dairy farms (6), whilst beef farms, in a region of high density of cattle and pig farms, were the most affected farms during the 2010 epidemic in Japan (10). Yang et al. (11) observed that high density areas of pig herds were the most affected in the 1997 FMD epidemic in Taiwan. The variable most strongly correlated with the spatial risk of outbreaks during the 2001 FMD epidemic in Argentina was cattle herd density (12). Similarly, it was shown that high farm density areas in The Netherlands would remain high risk areas if EU standard interventions are taken during a FMD epidemic (13). The relevance of farm density was also considered during the localized FMD outbreaks detected in UK in 2007. This minor epidemic involved eight outbreaks. Analysis of this epidemic led to the conclusion that the low density of cattle herds of the affected area combined with effective control measures, may explain why the virus did not spread more widely (14). In addition, some variables related with indirect transmission were reported as FMDV risk factors in the 2007 FMD epidemic in the UK. The risk of inadequate biosecurity was measured by indicators such as: location of car parking in relation to livestock areas, the presence of gates or physical barriers and whether farms were located next to public roads (14). Likewise, during the 2010 FMD epidemic in Japan (10) it was observed that physical barriers were found to be a protective factor, whereas the movement of people, vehicles and farm equipment were risk factors.

Whilst FMD epidemics have been extensively studied in developed countries, epidemics in developing countries have been sparsely studied. Here we address this limitation by systematically studying the historic 2001 epidemic in Uruguay. This paper aims to contribute to the knowledge of the epidemiology and transmission of FMDV in extensive production systems. The objective of this study was to identify the risk factors associated with infection and spread of the virus within the country.

## 2. Materials and methods

### 2.1. The epidemic

The description of the epidemic was provided by the Epidemiology Department of the Official Veterinary Services of the Ministry of Livestock, Agriculture and Fisheries of Uruguay (15).

On 23 April 2001, a suspicion of FMD occurrence was reported. The Veterinary Services investigated and clinically confirmed the disease on 24 April 2001. The official laboratory (DILAVE) confirmed the diagnosis by serology, identifying antibodies against type A virus on 25 April 2001. On 3 May, serotype A was confirmed by The Pan American Center for Foot-and-Mouth Disease (PANAFTOSA) (16).

The index case was confirmed in Soriano which is a Department located southwest of Uruguay at the border with Argentina. The most probable hypothesis established by the Official Veterinary Services was that the FMDV was introduced by fomites from Argentina. This hypothesis was supported by the fact that the virus type identified was the same in both countries and no live animal movements from Argentina to Uruguay were observed during or shortly before the high-risk period (HRP), which is the period between the virus introduction and the index case detection. The border administrative departments of Soriano and Colonia (southwest of Uruguay) involve intense cross-border movement of tourists, hunters and people related to the agriculture sector.

First, authorities sought to control the disease without vaccination, implementing measures such as ban of animal, animal products and people movements, culling the clinically affected and in contact FMDV susceptible animals within the outbreak and the application of biosecurity measures. In addition, a ring vaccination within a 10 km radius around outbreaks was implemented on 26 April. The ban of animal movements was enforced locally for 3 days, but then on 27 April 2021 this measure was extended at a national scale. On 30 April the measures changed, stamping out was stopped and emergency vaccination within an extensive buffer zone was enforced, which aimed to stop the FMDV spread from southwest to northeast. Later in the epidemic, infection appeared to keep spreading and on 5 May 2001 a mass vaccination of the entire national cattle population was adopted, which was completed on 7 June. The ban on animal movements was in place until June 7 after completing the first round of vaccination. Animal movements were authorized under certain defined conditions and requirements, which were lifted after completing the second round of vaccination. The second round of mass vaccination was implemented from 15 June to 22 July 2001.

**Figure 1:**
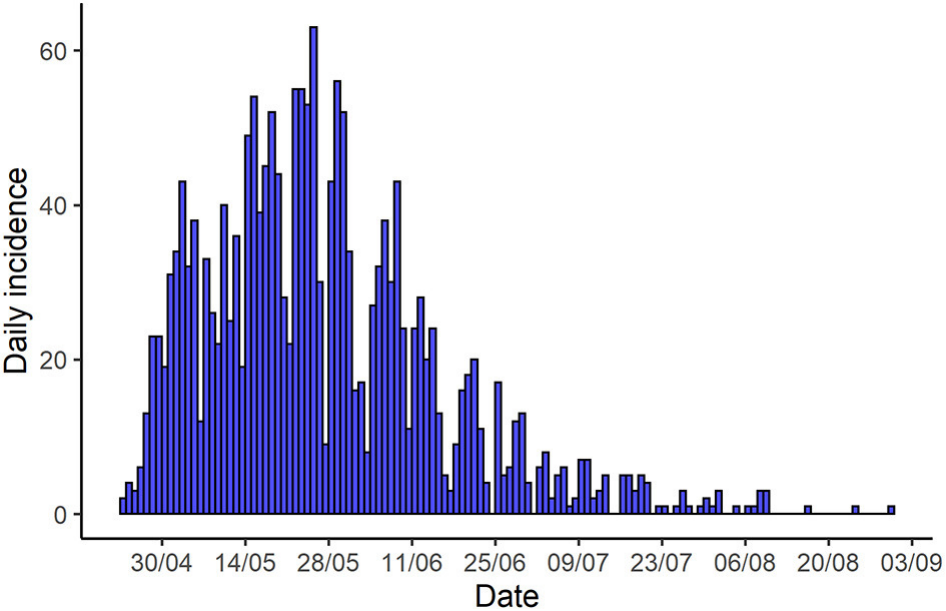
Time series of the number outbreaks (farms) detected per day in the 2001 epidemic by date of report.

The epidemic duration was 119 days, with the last outbreak being reported on 21 August 2001. At the end of the epidemic, the total number of FMDV infected farms (outbreaks) was 2,057. The epidemic curve, i.e., the distribution of outbreaks over time, has a steep increase and a long tail. The number of newly detected outbreaks increased from the first outbreak until the 25 of May, when the number of detected outbreaks reached the peak (65 farms/day). The declining phase was longer than the initial growth and reached a steady one to eight outbreaks per day for 2 months from 29 June (Figure 1).

### 2.2. Data source

In this study, the start of an FMDV epidemic was defined as an infected herd officially diagnosed either clinically or serologically. During the epidemic, the Veterinary Services gathered the information for each infected herd. It consisted of a farm unique identifier, the geographical location, and farm's stock and production information, such as animal numbers, animal species kept, the number of animals identified as infected by clinical inspection per species present, and the day of FMD/FMDV detection in the farm. The data also contained an assumed day of the onset of symptoms for each infected premise, which was estimated by the veterinary officer during the outbreak visit. These official reports were merged with the data on the location and livestock composition for all Uruguayan farms that kept cattle or sheep in 2001 (DICOSE Annual Affidavit, 2001, Curtesy of The Ministry of Agriculture). Only those records that had complete information were considered for the analysis.

### 2.3. Data analysis

#### 2.3.1. FMDV introduction model

Preliminary investigation of the available data indicated some limitations. One was uncertainty about a more exact date of onset of symptoms in each farm based on age of FMD lesions. For several reports onset of symptoms was reported as the first day of the visit to an infected farm which was unlikely as there were already large numbers of clinically infected animals. This observation may indicate a longer period of infection in the farm than was reported. Therefore, a mathematical model was developed to estimate each farm's infection dates.

A simple stochastic susceptible-exposed-infectious-removed (SEIR) model considering only the cattle population was used to describe the transmission of FMDV within herds (17–20). The model and its parameters are presented in Supplementary material S1 and Supplementary Table S2.

For simplification, we only considered the cattle population based on the fact that cattle was the most affected species during this epidemic (attack rate cattle: 5%, attack rate sheep: 0.02%). The stochastic model was performed for six scenarios that were set based on three different farm sizes of dairy and beef farms. By using these scenarios, the difference in the within farm transmission of FMDV between dairy and beef production systems was considered. Subsequently, we explored the time distribution for different accumulated numbers of clinical infectious bovines (CI), since the goal was to estimate the time of FMDV introduction based on the number of diseased animals. Finally, the mean time for each scenario was used to create general rules that were applied to the dataset to estimate each farm's infection dates. These analysis were performed in R version 4.0.4 (21).

#### 2.3.2. Identification of risk factors

For this part of the study, the epidemic was divided into four periods in terms of the control measures implemented: (1) The high-risk period (HRP) corresponded to the period between the FMDV introduction and the index case detection when control measures were not yet taken. (2) The local control measures period (LCM) referred to the period between the first outbreak detection and the mass vaccinations implementation. (3) First (dose) mass vaccination round and (4) Second (dose) mass vaccination round.

First, the association between the FMD status of the farms (infected/not infected) and potential risk factors was assessed using a multivariable logistic regression model for the HRP. Our model building and variable selection approach started from formulating a hypothesis based on prior knowledge of FMD epidemiology, followed by careful control for confounders. To control confounding, we mainly considered the biological interpretation taking into account the causal structure underlying the hypothesis. First, an univariable analysis was performed and variables with a *P*-value < 0.20 were eligible for inclusion into the multivariable model. The assumption of linearity of continuous variables included in the analysis (herd density, farm size, and cattle young stock proportion) was assessed by plotting the midpoints of the quartiles vs. the logit for infection. To account for deviations in linearity, natural cubic splines or categorization on continuous variables were used. Additionally, biologically significant variables were evaluated as effect modifiers and were retained in the model if the *P*-value was <0.05. Variable significance was assessed using the Likelihood Ratio Test (LRT). The overall fit of the model was evaluated by the Hosmer–Lemeshow goodness-of fit test (22) and the area under the ROC (Receiver Operating Characteristic) curve. Correlation between continuous variables were examined using the Pearson's coefficient, while chi-squared were used to assess the relation between categorical predictors.

The following eight variables were selected to build the multivariable model for the HRP: (1) cropping farms as a categorical variable (no/yes: farms that combine livestock production with cropping); (2) herd density as a continuous variable [total number of farms divided by the police section area^1^ (in km^2^)]; (3) farm size representing the number of cattle and sheep as a categorical variable (<36/36–134/134–463/>463); (4) species as a categorical variable (mixed: cattle & sheep/cattle only sheep only); (5) animal movements as a categorical variable (no/yes); (6) the distance from farms to main routes as a categorical variable (<100/≥100 m); (7) the presence of pig as a categorical variable [no/family (<19 pigs)/company (>19 pigs)]; and (8) the geographic coordinates (*x* and *y*) as natural cubic splines. Interaction terms between herd density and farm size, cropping and species, cropping and farm size, cropping and pigs, species and farm size, species and pig, pig and farm size were also investigated.

A multivariable model including the same variables as the HRP model was applied for each of the epidemic periods: LCM, first and second round of mass vaccination.

Finally, we created a subset of data to explore the risk factors associated with infection in those farms with at least one bovine i.e., cattle only herds and cattle and sheep herds. We considered two extra variables for this model: (1) the production system including beef, dairy and mixed categories, and (2) the proportions of cattle under 2 years of age as a continuous variable. A higher proportion of young stock (cattle between 12 and 24 months) in a herd was previously reported as high risk of increasing the susceptibility to infection by FMDV (14, 20).

All analysis were conducted in R version 4.0.4. by using the following main packages: “car,” “ROCR,” “rms,” “splines,” and “tidyverse” (21, 23–26).

## 3. Results

### 3.1. Summary description of the data

We analyzed a dataset with 1,867 outbreaks (farms) and 43,068 uninfected farms. Although 2,057 farms were affected during the epidemic, we considered only those records that had complete information. The dataset only considered farms that had at least one cattle or one sheep, i.e., it was not including those farms that raised only pigs. Farms that kept pigs only were not affected during the 2001 FMD epidemic.

In Uruguay, in 2001, there were more than 10.7 million cattle in 43,724 farms, around 12 million sheep in 25,594 farms, and almost 100,000 pigs in 3,461 farms (DICOSE Annual Affidavit, 2001, Curtesy of The Ministry of Agriculture). Most of the farms produced beef with cattle raised on grazing land under natural conditions. Generally, this type of farms (beef) raised cattle and sheep (mixed). Pigs were less relevant in terms of number of animals and farms (Table 1).

**Table 1:** Number of outbreaks and uninfected farms during the entire epidemic considering the species kept, the farm size distribution and the production system within them.

**Species**	**Outbreak**	**Number of farms**	**Farm size med (IQR)**	**Number of animals**	**Production system (farms)**		
**Cattle**					**Beef**	**Dairy**	**Mixed**
	Yes	1,859	491 (199–969)	1,768,054	1,519	170	170
	No	41,865	65 (21–201)	8,951,367	37,353	3,024	1,488
**Sheep**					**Mixed** ^a^	**Sheep only**	
	Yes	1,145	449 (113–1,243)	1,149,094	1,138	7	
	No	24,449	159 (50–444)	11,000,000	23,246	1,203	
**Pig**					**Family**	**Company**	
	Yes	190	10 (4–23)	4,341	134	56	
	No	3,271	7 (3–19)	89,053	2,468	803	
Total farms^b^	Yes	1,867		2,921,489			
	No	43,068		20,040,420			

a When we considered the species kept by farms there are three categories: mixed, sheep only and only cattle. In the table cattle only is not shown because it overlaps with production system (beef, dairy, and mixed). Only cattle: num. of farms: (yes = 722, no = 18,619).

b The sum of the number of farms that keep each species do not match the total farms because there were mixed farms that kept more than one species.

During the epidemic 1,861 farms presented clinical diseased cattle, whereas only six and nine farms also showed diseased sheep and pigs, respectively. On the other hand, clinical disease was detected in all species (cattle, sheep, and pig) only in one farm.

The proportion of the variables of interest among outbreak farms and uninfected farms for each epidemic period are presented in Supplementary Tables S9–S12.

### 3.2. Time of introduction of infection in each farm

**Figure 2:**
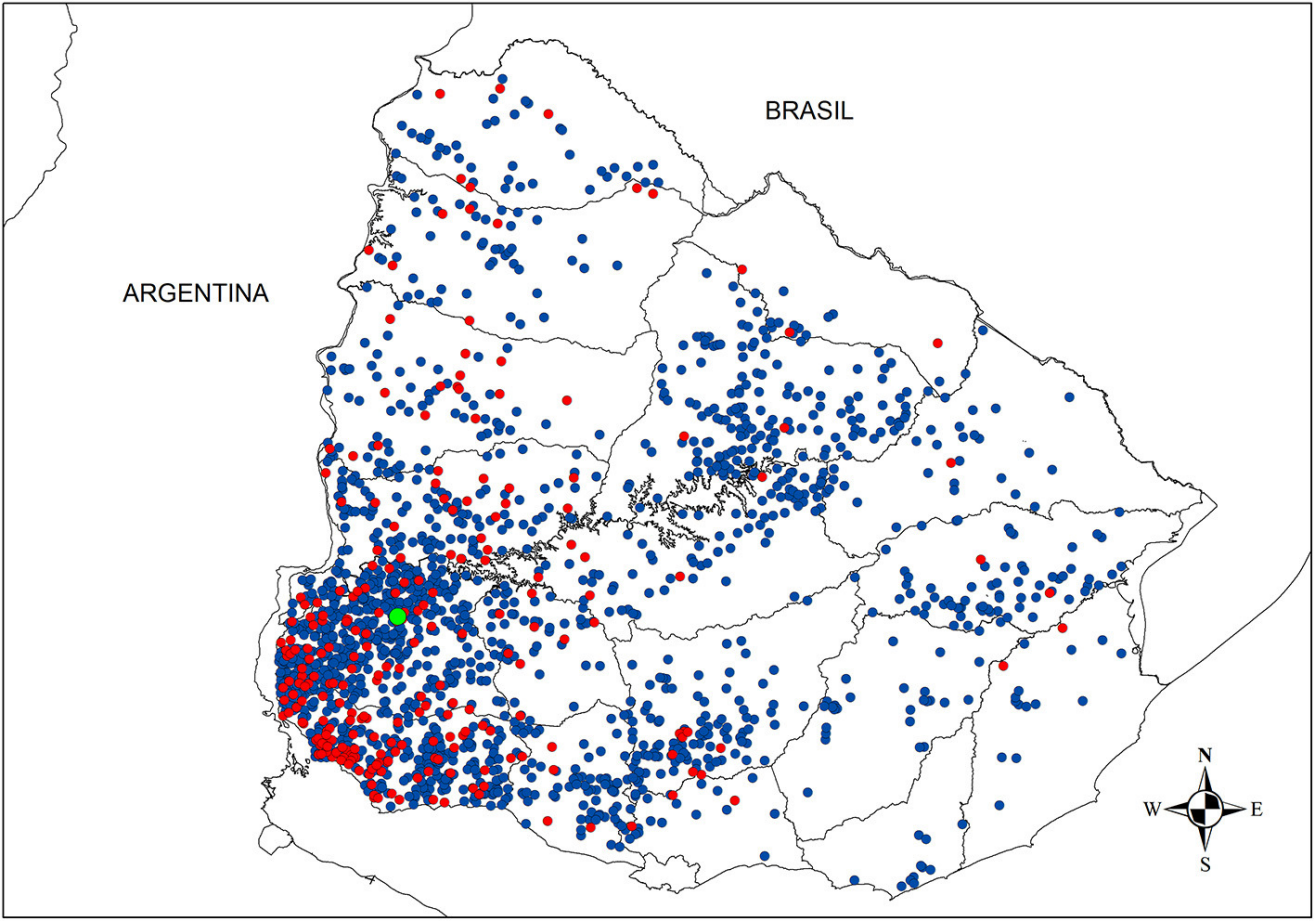
Spatial distribution of outbreaks. Farms that were infected during the high-risk period (HRP) are represented by red dots, while those infected in the following periods are showed in blue. The HRP corresponds to the period between the FMDV introduction and the index case detection (illustrated by a green dot).

We created general rules by using the results of the FMDV introduction model that were applied to the original dataset to estimate each farm's infection date (Table 2). The results of the FMDV introduction model for each scenario are presented in Supplementary Tables S3–S8. These new estimated approximate dates of infection were applied to the dataset, and outbreaks were assigned to four consecutive epidemic periods (Table 3). The results showed that probably around 242 farms were already infected by the time the first outbreak was confirmed (Figure 2).

**Table 2:** General rules created by using the results of the FMDV introduction model, which were used to estimate approximate each farm's infection dates. A simple stochastic susceptible-exposed-infectious- removed (SEIR) model considering only the cattle population was used to describe the transmission of FMD virus within herds.

	**Estimated time of FMDV introduction (days before recorded visit)**	

**Number of diseased animals**	**Dairy**	**Beef**
< 5	7	8
5–15	10	10
15–39	12	12
>39	13	14

**Table 3:** Number of outbreaks and uninfected farms for each epidemic period in Uruguay in 2001, including its duration and the number of outbreaks per day.

	**HRP**	**LCM**	**FirstVAC**	**SecVAC**
Uninfected farms	44,693	44,306	43,205	43,068
Outbreaks	242	387	1,101	137
Duration (days)	15	10	33	44
Outbreaks/day	16	39	33	3

HRP, the high-risk period corresponds to the period between the FMDV introduction and the index case detection; LCM, the local control measures period refers to the period between the first outbreak detection and the mass vaccinations implementation; FirstVAC and SecVAC are the first (dose) mass vaccination round and second (dose) mass vaccination round.

### 3.3. Risk factors for FMDV infection

We first analyzed the HRP; the results of the univariable analysis are presented as Supplementary Tables S9–S12. The following six variables were kept in the model for the HRP: (1) cropping; (2) herd density; (3) farm size; (4) species; (5) animal movements; (6) the geographic coordinates (*x* and *y*). The variables “distance from farms to main routes” and the presence of pig were removed from the model because there was no significant evidence of the contribution of these factors to the farm infection status. Only the interaction between herd density and farm size was considered in the model due to its epidemiological importance even though its level of significance (Chisq = 7.703, Df = 3, *p* = 0.053) was at the limit of the significance threshold *p* < 0.05.

**Figure 3:**
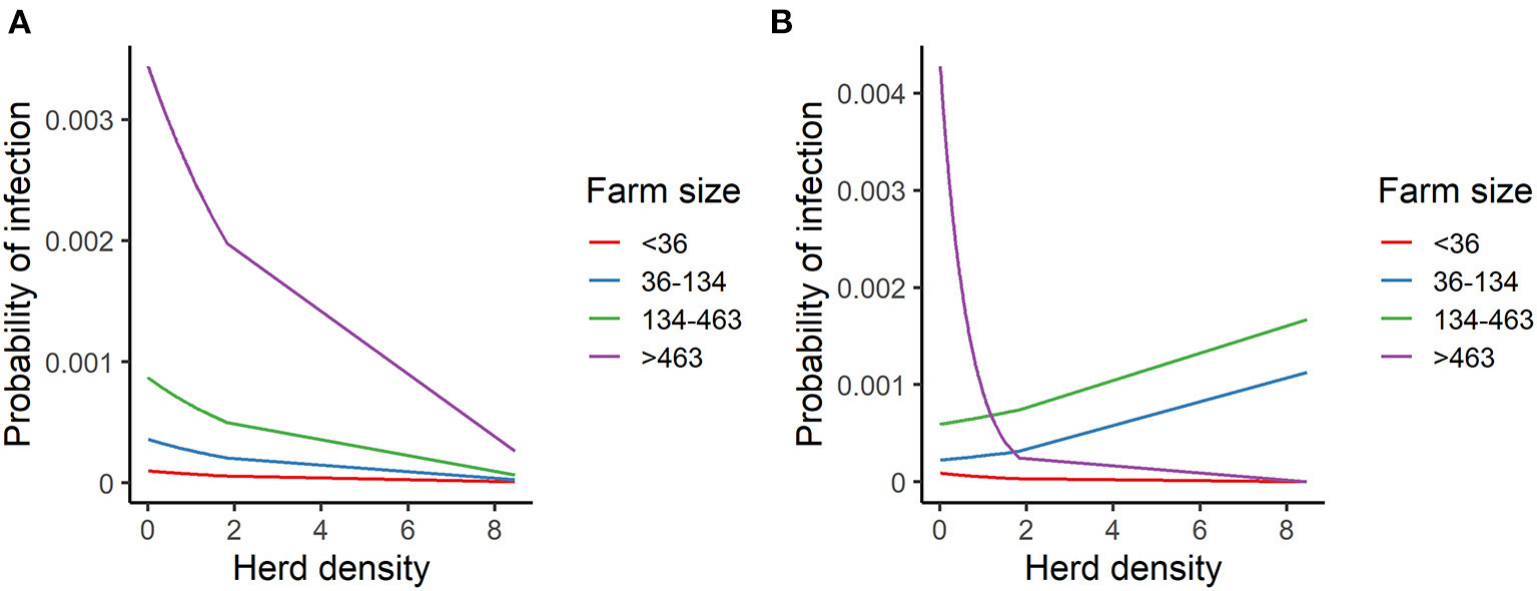
Predicted probability of FMDV infection for herd density across the farm size strata. **(A)** Model without interaction. **(B)** Model with interaction.

The odds ratio (OR) of being infected with FMDV for each variable after controlling for all variables included in the model for the HRP are presented in Table 4. The odds of being infected was higher for larger farms compared to small farms, and the size of this difference is dependent on the herd density of the area that the farms were located. It can be seen that, if the interaction between these variables is not considered, the probability of infection decreases for each farm size strata as herd density increases (Figure 3A). However, by including the interaction term between herd density and farm size, the direction of the relationship between the probability of infection and herd density changes for the intermediate strata (36–134 and 134–463), showing a higher probability of infection as farm density increases (Figure 3B). On the other hand, for large farms the probability of being infected decreases as herd density increases.

**Table 4:** Multivariable logistic regression output (OR: odds ratio) for the high-risk period (HRP).

**Predictors**	**Odds ratios**	**CI (95%)**	**Pr** ** (Wald)**	**Pr** ** (LRT)**
(Intercept)	0.02	0.00–0.09	< 0.001	
Cropping				< 0.001
Yes	1.93	1.43–2.60	< 0.001	
Herd density	0.57	0.03–1.71	0.671	0.412
Farm size				< 0.001
36–134	2.46	0.46–10.74	0.3	
134–463	6.53	1.27–27.98	0.029	
>463	48.56	9.39–210.18	< 0.001	
Species				0.042
Cattle	1.24	0.90–1.70	0.177	
Sheep	0	0	0.96	
Movements				0.001
Yes	1.57	1.19–2.06	0.001	
Latitude				< 0.001
ns1^a^(lat_y)	0.02	0.00–0.09	< 0.001	
ns2^a^(lat_y)	0.11	0.04–0.27	< 0.001	
Longitude				< 0.001
ns1^b^(lon_x)	0.0003	1.03E-04– 9.59E-04	< 0.001	
ns2^b^(lon_x)	0.03	0.004–0.12	< 0.001	
Interaction				0.053
hd_km2:Farm_size (36–134)	2.11	0.38–47.61	0.575	
hd_km2:Farm_size (134–463)	1.97	0.33–45.01	0.615	
hd_km2:Farm_size (>463)	0.36	0.05–9.04	0.473	

Hosmer–Lemeshow GOF test: *X*-squared = 2.904, df = 8, *p*-value = 0.940.

Area under ROC: 0.9258.

a Knots for natural spline (ns) effects with 2 degrees of freedom [ns(latitude, df = 2)]: 6,287,201.

b Knots for natural spline (ns) effects with 2 degrees of freedom [ns(longitude, df = 2)]: 487,821.

**Figure 4:**
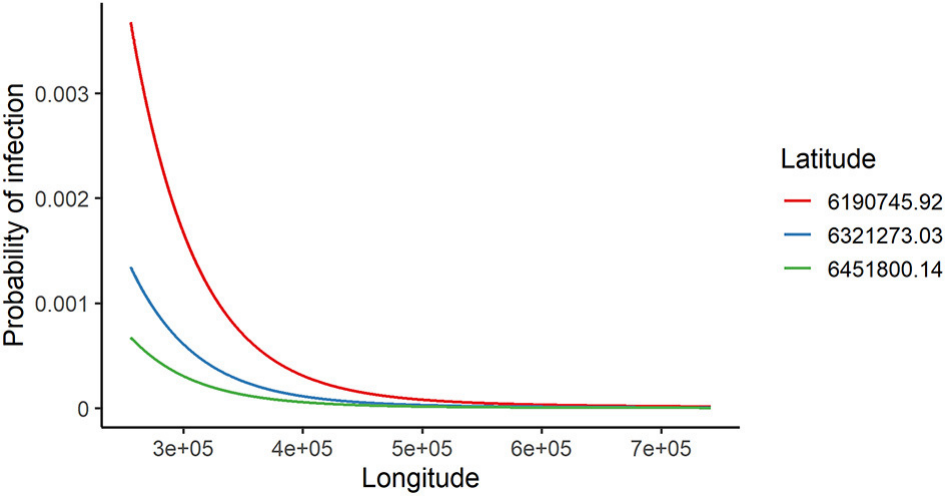
Predicted probability of FMDV infection during the high-risk period (HRP) vs. geographic coordinates. Longitude increases from east to west, and latitude increases from south to north.

The odds of being infected for farms that combined livestock with crop production was 93% higher compared to those that did not have cropping as an activity. The odds of being infected was 24% higher for farms that raised cattle only compared to mixed farms (cattle and sheep). On the other hand, the chance of infection was almost zero for farms that raised sheep only compared to mixed farms. Interestingly, those farms with animal movements had 57% higher odds of infection than those farms without animal movements. Finally, the risk of FMDV infection was higher in the southwest and it decreases as longitude and latitude increase, from west to the east and from south to north, respectively (Figure 4).

**Figure 5:**
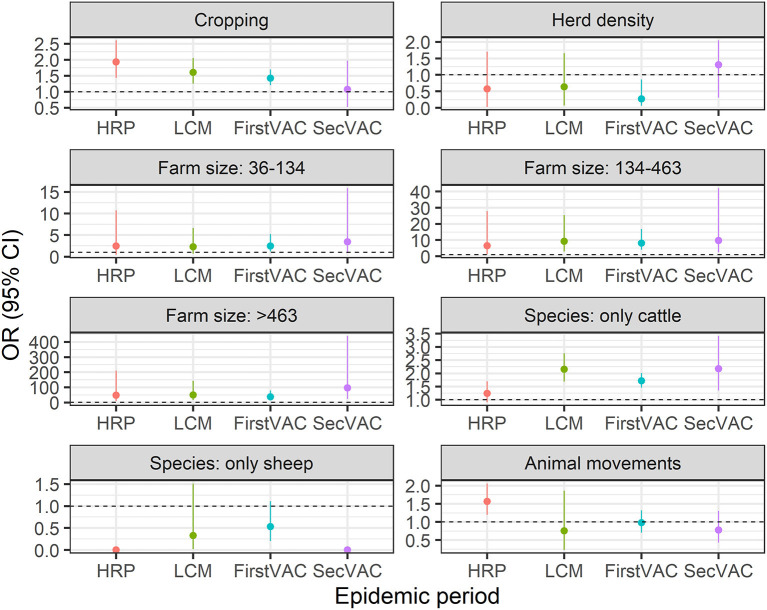
Odds ratio (OR) of each explanatory variables for consecutive epidemic periods. HRP, the high-risk period; LCM, local control measures period; FirstVAC and SecVAC are the first (dose) mass vaccination round and second (dose) mass vaccination round. Reference levels for categorical variables are: (1) Cropping: Farms that did not combine livestock with crop production; (2) farm size: <36 animals; (3) species: Farms that kept cattle and sheep (mixed); (4) animal movements: Farms without animal movements recorded. Regarding farm size variable, the estimated OR of “36–134” category was not significant for the HRP, LCM, and SecVAC periods, whereas was significant for the entire epidemic for categories “136–463” and “>463”.

For the following three periods of the epidemic multivariable models including the same variables as the HRP model were applied and the results are graphically shown in Figure 5. The findings indicated that animal movements were significantly associated with FMDV infection only during the HRP. Large farms and farms that kept cattle only remained as risk factors during the whole epidemic. Cropping farms remained at high risk over the HRP, the LCM and first vaccination periods. Finally, the risk of infection continued to decrease as the latitude increased (from south to north) throughout the entire epidemic, while for longitude (from east to west) the same effect was observed until the first vaccination period.

**Figure 6:**
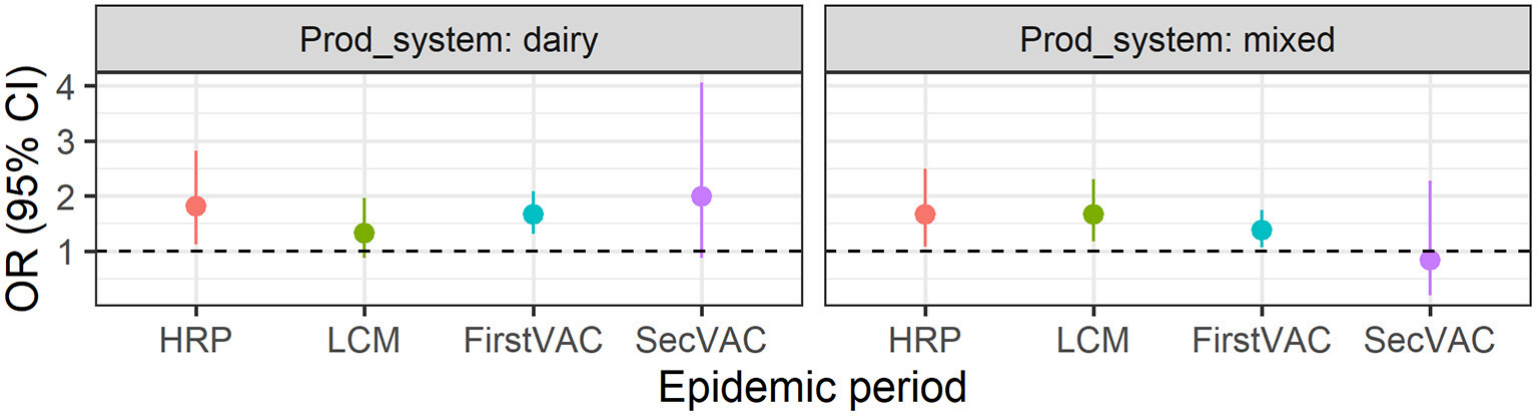
Odds ratio (OR) of production system being “beef” the reference, after controlling for all variables included in the model for each epidemic period. HRP, the high-risk period; LCM, local control measures period; FirstVAC and SecVAC are the first (dose).

As cattle farms were at higher risk of being infected, we fitted a model using only data from cattle farms for which we considered two extra variables: (1) the production system as a categorical variable including beef, dairy and mixed (beef and dairy) categories, (2) the proportion of cattle under 2 years of age as a continuous variable. Both variables were significantly associated with farm FMD status in an univariate analysis. However, only the production system remained significant after controlling for other variables in the multivariable logistic regression model. During the HRP, the odds of being infected was 81% higher for dairy farms [OR = 1.81 (95% CI: 1.12–2.83)] and 66% higher for mixed farms [OR = 1.66 (95% CI: 1.08–2.49)] compared to beef farms. Moreover, dairy and mixed farms remained at high risk of infection until the first vaccination period (Figure 6).

## 4. Discussion

The objective of this study was to identify risk factors associated with the becoming an FMDV outbreak on farms during the epidemic in Uruguay in 2001.

We acknowledge some limitations. First, the date when the FMDV infects each herd, on which all epidemiological outputs were based, was not available for this epidemic. Our estimation of these dates was based on the number of cattle showing FMD-like clinical signs rather than the number of truly infected animals. The veterinary officer recorded the number of clinical diseased animals during the first visit of each infected farms. The need to implement the control measures as rapidly as possible, together with the risk of within herd transmission during animal inspection, may be the reason not to do a whole herd examination, leading to some inaccuracy in the estimation of the within herd incidence. Nevertheless, assuming that bias in this estimation were non-differential, the outputs of the analysis are unlikely to have been considerably biased. Moreover, if we had followed the approach used by Bouma et al. (6) in the analysis of FMD epidemic in the Netherlands in 2001 and assume that when only a few animals (<10) were diseased the infection started 1 week earlier; and when more than 10 animals were clinically affected the infection started 2 weeks earlier, we would have obtained similar results (data not shown). The diagnosis based on clinical signs raise another important point of discussion due to the difficulty in making clinical diagnosis in sheep as this species show less evident clinical signs when is infected with FMDV (27). However, the veterinary services of Uruguay carried out a sero-epidemiological survey at the end of the epidemic (August 2001) to estimate the prevalence of FMDV in sheep within the outbreaks, which showed a 1.9% prevalence of antibodies against FMDV infection-associated antigen (VIAA). In addition, they tested sheep, sampling farms by geographical strata based on distance from the nearest FMD outbreaks (between 5 and 10 km, and >10 km), which showed a 0.4 and 0.3% of antibodies, respectively. These results support the clinical diagnosis made by veterinary officers during this epidemic [Epidemiology Department (DGSG-MGAP), personal communication, 2021].

Our analysis presented here showed that 242 farms were likely already infected at locations spread throughout the country by the time the first outbreak was confirmed. This delay in the detection of the index case probably hampered the efficacy of the first locally implemented measures and played a key role in determining the development of a large epidemic in Uruguay. The movements of animals, animal products, vehicles and people at the time that FMDV was already introduced in the country, could have contributed to FMDV spread over long distances. A failure in early detection was reported in all other major epidemics that occurred in previously FMD-free countries (5, 8, 28, 29). Several factors can be related to the delay in the FMD detection such as the lack of awareness of clinical signs being the cause of suspicion of FMD in free countries, the need of high within herd prevalence to detect the disease, fear and discouragement of reporting, etc., illustrating a need to continuously improve surveillance and awareness systems.

During the HRP, the probability of being an outbreak herd was significantly associated with animal movements, farm size, the geographical location, and whether farms combined livestock with crop production. Some of these variables may be associated with the introduction of infection in the country. Since an FMD epidemic had been already ongoing in Argentina before January 2001 (8), there was no animal movement recorded between Argentina and Uruguay, and given that the virus type identified in Uruguay was the same as the one circulating in Argentina, it was hypothesized that the virus was introduced by fomites from this country. The illegal movement of animals from Argentina should be considered as other possible source for FMDV introduction. This pathway was less probably than the introduction from fomites since the border between Argentina and Uruguay consist of the Uruguay River, which functions as a natural barrier for the movement of animals. Our analysis provides some additional evidence to this hypothesis by confirming that those farms situated near the border were at higher risk of infection (higher risk observed in coordinates closer to Argentina) and by finding an association between infection and cropping activity. The latter reflects the intense cross-border movement during the HRP of people and cropping equipment and may provide an indication of indirect exposure of livestock in those farms to contaminated equipment.

Several researchers have reported high herd density as a risk factor associated with FMD (10–13). We found that the direction and the size of the association between herd density and the probability of infection depended on farm size. We observed that high herd density was associated with FMDV infection only for medium farm size (36–463 animals). In contrast, we observed higher probabilities of infection for large farms (>463 animals) which were situated in low farm density areas. This may reflect that in extensive livestock production systems, where cattle are mainly raised in grazing areas, farm size is much related to land size. Therefore, farms located in high farm density areas in Uruguay are mainly small farms (<36 animals), which did not contribute to FMDV transmission during this epidemic (<3% of the infected farm were small farms). On the other hand, large (>463 animals) and medium-size farms (134–463 animals) had been more affected during this epidemic. These kinds of farms may involve frequent movements of people, farm equipment and vehicles such as feed transport vehicles, which could be routes of FMDV transmission. A similar conclusion was reached in studies of the FMD epidemic in Japan in 2010 (10, 30). These results are also partially consistent with findings from studies of the epidemic in Argentina, which showed that the density of outbreaks was most strongly correlated with the distribution of large farms during and after the mass vaccination campaign (12).

The delay in the detection of FMD as well as in the enforcement of the nationwide livestock movement ban likely allowed infected animals to move between farms and to be traded through livestock markets, contributing to the transmission of FMDV. As expected, the movements of animals were no longer a risk factor after the HRP probably due to the control measures implemented, such as banning of movement and the official control and vehicles disinfections for those movements that were officially authorized. Further research is planned to study the role played by animal movements in the FMDV spread during the HRP.

We found that cattle farms were at higher risk of infection from the end of the HRP compared to mixed farms, which kept cattle and sheep, and sheep farms. This finding is consistent with that of de Rueda et al. (31), who indicated that in this kind of mixed populations sheep play a more limited role in the FMDV transmission compared to cattle. These authors found that infectivity of sheep is lower than infectivity of cattle, while their susceptibility to FMDV is similar.

Regarding the production system of cattle herds, dairy and mixed farms, which combined beef and milk production, remained at higher risk of infection than beef farms until the end of the first mass vaccination period. This is probably related to the fact that higher frequency of different contacts between dairy herds is expected such as lorries for milk collection, veterinarian's visits, farm equipment, people, feed transport vehicles, etc., leading to higher probabilities of transmission between these types of production. These results are in line with findings from the study of the 2001 FMD epidemic in the Netherlands (6) in which 23 of the 26 affected farms were dairy herds, and with the 2001 FMD epidemic in Argentina (12).

The presence of pigs in farms was not significantly associated with FMDV infection during the entire epidemic. Pigs are much less relevant in terms of the number of producers and animals in Uruguay compared to cattle and sheep. There were only around 100,000 pigs in Uruguay in 2001, while the cattle and sheep population stock were almost 10.6 and a 12.1 million heads, respectively (DICOSE Annual Affidavit, 2001, Curtesy of The Ministry of Agriculture). During the 2001 FMD epidemic in Uruguay, 112 pigs were affected in only nine of 2,057 outbreaks. Therefore, the effect of high virus excretion by pigs when they are infected with FMDV and their ability to infect other animal species (32), which was reported in the 2010 epidemic in Japan (28) could not be observed during this epidemic in Uruguay. The pig swill-feed practice could be a risk factor for introduction of FMDV and was the most likely source of infection of the index case in the 2001 FMD epidemic in the UK (5) and the FMDV type O introduction in Uruguay in 2000 (7). The feeding of swill might be more frequently in pig's familiar production systems (few animals) in Uruguay in 2000. However, we did not find an association between pig's familiar production systems (defined here as farms with <19 pigs) and FMDV infection during the 2001 epidemic after controlling for the other variables of interest.

Researchers that analyzed the 2007 outbreak in the UK concluded that infection of secondary farms appeared to be driven mainly by biosecurity and environmental risk (14). In our study, information regarding biosecurity measures implemented at herd level was not available and the assessment of risk factors related to fomite transmission was limited. However, we found a significant association between FMDV infection and the following factors such as cropping, farm size and production system that may indirectly indicate the contribution of lack of biosecurity, through indirect contacts, in FMDV transmission. We also explored the role of farm distance from main routes as a factor of increasing the risk in contact between FMDV and susceptible animals but it was not significant. This may reflect the effectiveness of the measures implemented after the detection of the index case. These measures consisted of the ban of animal movements, the disinfection of vehicle wheels at farm entrances and exits, and strategic points of disinfection on routes.

Large farms that kept cattle remained at higher risk of infection during the epidemic. Similarly to the epidemic in Argentina (12), this may reflect that it is more difficult to achieve effective immune protection in such large herds compared to small herds where it is easier to achieve full vaccination of the animals. Furthermore, this may indicate the need for second rounds of mass vaccination to control large epidemics in extensive management systems.

In conclusion, our results suggest that the 2001 FMD epidemic was large as a result of a combination of factors including the delay in the detection of the index case, the movements of infected animals during the HRP, more difficult vaccination in large farms and insufficient biosecurity measures at the herd level. Large farms that kept cattle remained as risk factors for the entire epidemic, suggesting that these types of farms would need more rigorous biosecurity measures to control future FMD epidemics, and should be targeted for surveillance in surveillance systems that aim to enhance early detection of FMDV infection.

The main practical contribution of our study is a better understanding of the risk factors associated with FMD infection of farms, which in combination to other epidemiological information provides support to inform surveillance and control strategies for future FMD epidemics.

## Data availability statement

The data analyzed in this study is subject to the following licenses/restrictions: Confidential database that contains exact farm location and other private information. Requests to access these datasets should be directed to unepi@mgap.gub.uy.

## Author contributions

JG, MJ, AG, and MI contributed to conception and design of the study. MI organized the database and wrote the first draft of the manuscript. MI and EFC performed the statistical analysis. All authors contributed to manuscript revision, read, and approved the submitted version.

## Footnotes

^1^The police section is an area that corresponds to the police precinct to which jurisdiction the farm is subject. It is used as the smallest administrative area in Uruguay.
